# Supplementary figures and images for: Predictors of Sarcopenia in Outpatients with Post-Critical SARS-CoV2 Disease. Nutritional Ultrasound of Rectus Femoris Muscle, a Potential Tool
Source: Nutrients. 2022 Nov 24;14(23):4988. doi: 10.3390/nu14234988 (PMC9740630; doi:10.3390/nu14234988)

**Supplemental Figure 1: Flow chart diagram of patients selection in our study.**

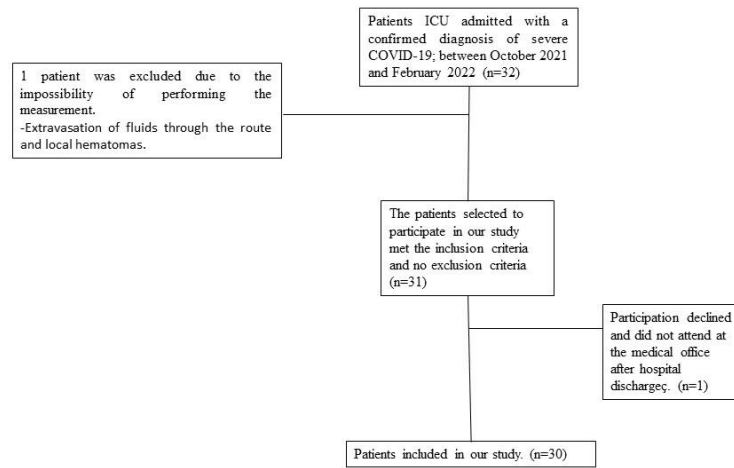

Supplement: Supplementary file 1 [file nutrients-14-04988-s001.zip › nutrients-2029398-supplementary.pdf]
